# Is more always better when it comes to mating?

**DOI:** 10.1371/journal.pbio.3001955

**Published:** 2023-01-11

**Authors:** Hanna Kokko, Michael D. Jennions

**Affiliations:** 1 Department of Evolutionary Biology and Environmental Sciences, University of Zurich, Zurich, Switzerland; 2 Ecology & Evolution, Research School of Biology, The Australian National University, Canberra, Australia

## Abstract

Fitness usually increases when a male mates with more females, but is the same true for females? A new meta-analysis in PLOS Biology shows that females, like males, tend to have a positive relationship between the number of mates and their reproductive output. This Primer asks why.

## Teaser

Fitness usually increases when a male mates with more females, but is the same true for females? A new meta-analysis in *PLOS Biology* shows that females, like males, tend to have a positive relationship between the number of mates and their reproductive output. But why?

Evolutionary biology offers insights for anyone interested in how diversity combines with general principles. Patterns exist [1] but often with numerous “exceptions to the rule.” One taxonomically widespread pattern that captured Charles Darwin’s attention is that females appear less interested than males in mating repeatedly with the same partner, or with multiple partners [2]. This was widely accepted as a fact, and in the ensuing decades, it became normal to refer to “coy” females and “eager” males, from birds to beetles. But there was no clear explanation for this asymmetry; worryingly, it also seemed to impose Victorian norms about gender roles in humans onto nonhuman animals [3,4].

The first data-driven general explanation for sex differences in selection for mating multiply involved experiments in the 1940s by Angus Bateman on fruit flies [5]. He showed the sexes can differ in the relationship between the number of mates and reproductive success. Whenever this relationship is steep, mating with more opposite sex partners is strongly linked with increased parental fitness, often interpreted as selection to mate multiply. This finding changed the tone of the conversation by providing a general principle that included the possibility that females are benefiting from multiple mating if they have a positive Bateman gradient. Fromonteil and colleagues [6] now show that positive female gradients are so common that they should be considered the norm, not the exception.

How does [6] change our view of nature? Recent evolutionary textbooks no longer describe females as “coy.” Instead, students are told that females often benefit from having multiple partners [3], as elegant experiments have shown that polyandry can elevate fitness [7]. Interestingly, our language to describe females has changed concomitantly with changing societal norms *and* accumulating data. Of course, the biology of nonhuman animals has stayed the same in recent decades, and science should uncover the truth and not change the story depending on what we happen to value societally at any given moment. This makes empirical data valuable. Fromonteil and colleagues [6] have added much needed generality with their meta-analysis of female Bateman gradients for 77 species. But how should we now characterize selection operating on a “typical” female?

Bateman gradients tend to be steeper in males than in females, which has previously been used to explain why males invest more in traits that increase their mating success (e.g., ornaments and weapons) [8]. But if both sexes have positive gradients, are females also typically under sexual selection and competing for mates? Fromonteil and colleagues favor this interpretation. To anyone disliking old sexual stereotypes, this is heartening. However, if there ever is a paper where one should read every sentence, not just the summary, it is this one.

There are many caveats in the text of [6], and for good reasons. Here, it is revealing to reflect on what was meant with the earlier usage of “coy.” One image invoked is a shy creature that blushes at the thought of more than one mate. The findings of Fromonteil and colleagues and others [7] definitively show that females typically have little trouble creating healthy broods with sperm from several males. Amusingly, mating with a single male (monandry) is therefore the unusual case that requires an explanation [9]! However, dismissing the image of blushing females is not the same as concluding that females are typically selected to elevate their mate acquisition rate.

Why does a positive Bateman gradient not straightforwardly imply that females must compete for males? One reason is the painful truism that correlation is not causation, and Bateman gradients usually rely on correlational data. Spurious correlations are a common pitfall should a third variable enter the scene. Consider the fact that fecundity often increases with female body size. Larger females will attract more males and have higher reproductive success and more mates than smaller females. If small females can still acquire enough sperm to fertilize their (smaller) clutches, a positive Bateman gradient will exist without any female having to exert effort finding mates. Positive correlations are thus compatible with no sexual selection on the sex with the shallower gradient, or even with scenarios where a slightly lower mating rate than observed would improve female fitness. So too, of course, are scenarios where mating more often *does* increase female reproductive output (e.g., when mating males provide their partners with food).

In our view, it is healthy to celebrate how much biological diversity in both sexes’ behavior can happily coexist with positive Bateman gradients. Alternative causal pathways, either alone or in addition to mating causally increasing reproductive output, can coexist within Fromonteil and colleagues’ findings. We worry that the focus in [6] on mate-limited females as the main pathway, while intriguing, is problematic. The extensive literature on sexual conflict [10] explains why there can be mate encounters where only one or both individuals (or several in a group setting) are keen to mate, while others are not. An optimal number of matings for females may, in many settings, reflect a trade-off between male-imposed costs (e.g., toxins in ejaculates) and diminishing gains from acquiring more sperm or other male-provided resources [10]. The ways to be disinterested in a particular mating are often much more active than any image of “coyness” could evoke: Weevil females kicking males to dislodge them is just one example.

If one only read [6] fleetingly, one might conclude that each additional mating further enhances reproductive performance in both males and females. We caution against this simplification and recommend rereading Fromonteil and colleagues’ Box 1. Should a reader forget the caveat that correlation is not causation, this leads to a surprisingly strong unintended consequence: a sudden inability to understand situations where some matings are “unwanted” by at least some individuals. Sexual conflict theory can explain this, and the data in [6] is equally compatible with a view where not every female strives to mate maximally often and where some females are not mate limited but already suffer from an overabundance of attention.

All this said, there is a sting in the tail. Are we being unfair to the authors? In [6], there is evidence that species with steeper female gradients are also those in which more females are polyandrous. Does this not imply that females generally benefit from multiple mating, these benefits therefore driving its evolution? Perhaps, but once again, the pattern is reconcilable with greater diversity. Some species may have females that receive “too much” male attention but manage to escape mating to a certain extent, while others are under selection to mate more. Polyandry levels and the associated gradients will then equilibrate at different points in each case. Distinguishing between hypotheses like this and [6]’s favorite interpretation remains a fascinating challenge.
